# Cutaneous xanthogranuloma associated with *Klebsiella* *pneumoniea* in a budgerigar (*Melopsittacus undulatus*)

**DOI:** 10.30466/vrf.2019.102621.2445

**Published:** 2019-12-15

**Authors:** Sara Shokrpoor, Amir Asghari Baghkheirati, Azam Yazdani, Jamshid Razmyar

**Affiliations:** 1 *Department of Pathology, Faculty of Veterinary Medicine, University of Tehran, Tehran, Iran; *; 2 *Department of Avian Diseases, Faculty of Veterinary Medicine, University of Tehran, Tehran, Iran.*

**Keywords:** Budgerigar, Histopathology, Polymerase chain reaction, Radiology, Xanthogranuloma

## Abstract

Budgerigar is a common name for a colorful Australian native bird belonging to the *Melopsittacus undulatus* species. It is a very familiar pet around the world and its breeding has been grown in Iran. This study was conducted on a 2-year-old budgerigar with a nodular mass on the left wing. Physical examination revealed a firm, round and well-circumscribed mass approximately 1.70 cm in diameter. Radiographs showed a soft tissue mass with no involvement of bony structures. Fine needle aspiration was performed and the sample was cultured. In cultural examination, *Klebsiella* spp. were isolated in pure culture. Genus and species of the bacteria were confirmed using multiplex polymerase chain reaction. The mass was surgically excised and it was mainly composed of numerous, large lipid-laden macrophages containing abundant vacuolated cytoplasm, extracellular acicular cholesterol clefts and large number of multinucleated giant cells (especially multinucleated Touton giant cells) in the dermis. Finally, a diagnosis of cutaneous xanthogranuloma was made based on histopathological findings.

## Introduction

Budgerigar is one of the most popular ornamental bird breeds**.** This tiny bird is gregarious and usually monogamous, however, there are individuals with polygamous tendencies.^1^

The xanthogranuloma is a degenerative lesion occurring through a nodular accumulation of cholesterol and other lipids accompanied by granulomatous inflammation (foamy macrophages and multinucleated giant cells).^2^ In birds, cutaneous xanthogranulomas are typically observed in the cervical region, wattles, back, distal wings, sternum, feathered skin over the tibial region or around the uropygial area.^3^ Xanthogranulomas have been identified in a variety of avian species including fledgling great horned owl (*Bubo virginianus*),^3^ chicken (*Gallus gallus domesticus*),^4^ American kestrel (*Falco sparverius*),^5^ red-tailed hawk *(Buteo jamaicensis*),^6^ blue and gold macaw (*Ara ararauna*),^7^ cockatiel (*Nymphicus hollandicus*),^8^ great white pelican (*Pelecanus onocrotalus*)^9^ and Egyptian Swift pigeon (*Columba livia*).^10^

*Klebsiella pneumoniae *as a non-motile, late lactose fermenting, oxidase-negative and rod-shaped bacillus with a polysaccharide-based capsule is a member of the Gram-negative Enterobactriacea family. This bacterium is a common saprophyte in many parts of the environment.^11^ To our knowledge, cutaneous xanthogranuloma associated with *Klebsiella *spp. in budgerigar (*Melopsittacus undulatus*) has not been reported previously.

## Case Description

**Fig. 1:**
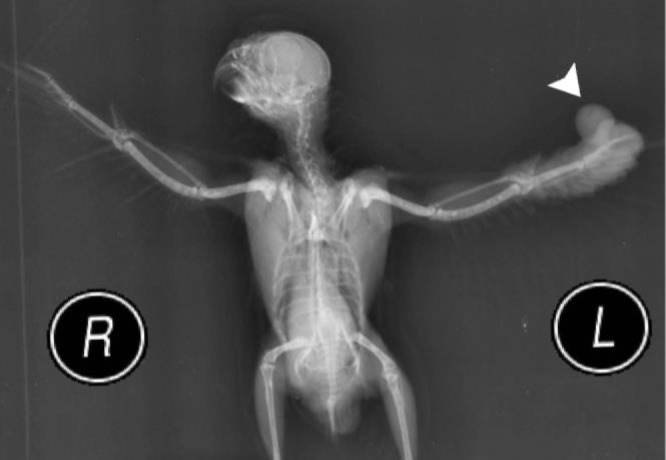
The mass (arrowheads) on the left wing of a budgerigar

A 2-year-old budgerigar, weighing 33.00 g, with a nodular mass on the left wing was referred to the Faculty of Veterinary Medicine, University of Tehran, Tehran, Iran (Fig. 1). The mass was firm, round and well-circumscribed. The overlying epithelium had ulceration and was not covered with the feathers. No other abnormalities were noted in physical examination. Standard whole body digital radiographs (DirectView Classic CR System; Kodak, Rochester, USA) were taken in left lateral and ventrodorsal projections. Bacterial cultures from the mass were also performed. The isolate was subjected to a multiplex poly-merase chain reaction (mPCR) to confirm genus and species of the isolate as described previously with modifications in annealing time and temperature.^12^^,^^13^ Briefly, the PCR reaction mixture consisted of 5.00 μL template DNA, 12.50 μL Master Mix RED kit (Ampliqon, Odense, Denmark), 1.00 μL (10.00 pmol μL^-1^) of each primers and distilled water to the final volume of 25 μL. Thermal cycling was carried out at 95.00 ˚C for 3 min followed by 35 cycles of 94.00 ˚C for 30 sec, 56.00 ˚C for 30 sec, 72.00 ˚C for 1 min and a final extension at 72.00 ˚C for 10 min. Then, PCR products were stained and visualized. All reactions contained positive control’s *K. pneumoniae *and *K. oxytoca* from previous work^13^ and distilled water was used as a negative control.

Finally, surgical removal of the mass was elected. The bird was anesthetized with 5.00% isoflurane, intubated and maintained on 2.00% isoflurane. Oxygen flow rate was 1.00 L per min. The mass (1.70 × 1.50 × 0.70 cm) was surgically excised. Its cross-section was comprised of a yellow and friable tissue with interspersed fibrous areas. Surgery was performed successfully and the bird was recovered uneventfully. Enrofloxacin (Science Laboratories, Tehran, Iran; 10 mg kg^-1^, twice daily for 5 days) was administered orally. The mass was placed in 10.00% neutral buffered formalin for histopathological evaluation and routinely processed, dehydrated, embedded in paraffin wax, sectioned at 5.00 μm in thickness using rotary microtome (RM2 145; Leica, Wetzlar, Germany) and stained with Hematoxylin and Eosin (H & E). Based on the owner information, the mass did not recur during the following three months.

## Results

**Fig. 2:**
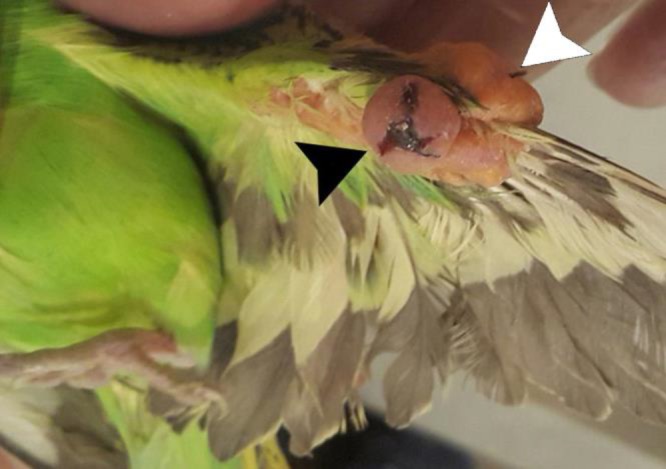
A soft tissue mass (arrowhead) in the distal aspect of the left wing was observed in radiography

Radiographs showed a soft tissue mass in the distal aspect of the left wing with no involvement of bony structures (Fig. 2). Bacterial cultures from the mass were carried out on Blood/MacConkey’s agar aerobically at 37.00 ˚C for 24 to 48 hr. Bacterial isolates were identified as *Klebsiella *spp. based on colony’s morphology, Gram staining and related biochemical tests. The presence of *Klebsiella pneumonia* was confirmed with mPCR assay at genus and species levels, in which the 441 bp amplicon represented *Klebsiella* spp. and 108 bp amplicon indicated *K. pneumonia.* The lack of 343 bp amplicon showed the absence of *K. oxcytoca* comparing to controls.^12^

**Fig. 3:**
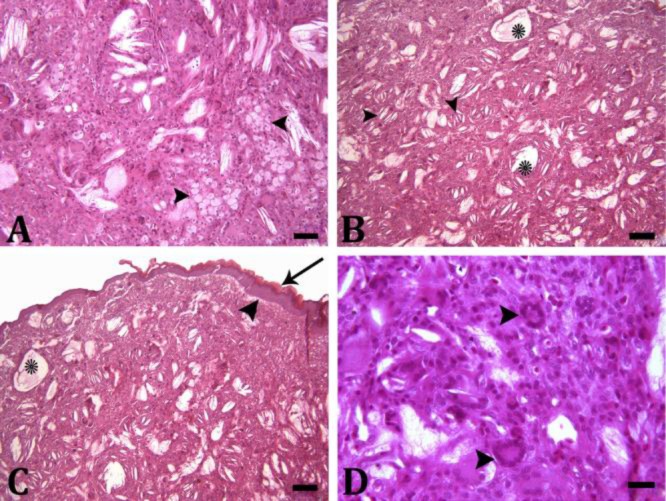
**A)** The mass with numerous, large lipid-laden macro-phages containing abundant vacuolated cytoplasm with an eccentric nucleus (arrowheads); **B)** Cyst-like aggregations of free lipid-droplets (asterisks) and extracellular acicular cholesterol clefts (arrowheads); **C)** Acanthosis (arrowhead), hyperkeratosis (arrow) and cyst-like aggregation of free lipid-droplets (asterisks); **D)** Multinucleated Touton giant cells (arrowheads) in the dermis, (H & E, Scale bars = 50, 100, 100, 20.00 µm, respectively)

Histopathologically, encapsulated mass was mainly composed of numerous, large lipid-laden macrophages containing abundant finely vacuolated cytoplasm with an eccentric nucleus (Fig. 3A). Cyst-like aggregations of free lipid-droplets in variable sizes and extracellular acicular cholesterol clefts were also observed (Fig. 3B). 

In some areas, mild acanthosis, hyperkeratosis and ulceration were also seen (Fig. 3C). A large number of multinucleated giant cells (especially multinucleated Touton giant cells) was also present in the superficial and deep dermis (Fig. 3D). Moderate number of lymphocytes was scattered throughout the mass. The histo-pathological findings supported a final diagnosis of cutaneous xanthogranuloma.

## Discussion

Xanthogranulomas are benign nodular granulomatous lesions occurring in the skin, subcutaneous tissues, tendons and internal organs of several species including birds.^7^ They are generally friable, yellow-colored and fatty appearing masses.^14^ As in the present report, xantho-granulomas are characterized by accumulation of lipid-laden macrophages, multinucleated giant cells and cholesterol clefts.^8^ In previous studies, similar to present case, xanthogranulomas were associated with fewer numbers of lymphocytes.^15^ Avian xanthomatosis is a condition of uncertain etiology. Abnormal lipid metabolism is thought to contribute to the xantho-granulomas formation and elevated serum cholesterol or other lipids that are present in affected individuals. In birds, hypercholesterolemia and hypertriglyceridemia have been reported in a blue and gold macaw (*Ara ararauna*) affected by a conjunctival xanthogranuloma and a goose (*Anser anser*) diagnosed with xanthomatosis.^7^^,^^15^ In this case, hematology and serum chemistry weren’t performed; thus, cholesterol level of the budgerigar is unknown. Cutaneous xanthogranulomas tend to develop in areas where physical trauma, local pressure, bleeding or inflammation have occurred or are occurring. In the present case, the mass was ulcerated and *Klebsiella *spp. were identified in the bacterial culture. On the basis of macroscopic and microscopic characteristics, origin of this xanthogranuloma remained unknown. However, ulceration can be a predisposing factor in xantho-granuloma development in the present case. Similarly, the surgical treatment of subcutaneous xanthogranuloma has generally had a favorable outcome.^8^
